# The ALBA spectroscopic LEEM-PEEM experimental station: layout and performance

**DOI:** 10.1107/S1600577515003537

**Published:** 2015-04-09

**Authors:** Lucia Aballe, Michael Foerster, Eric Pellegrin, Josep Nicolas, Salvador Ferrer

**Affiliations:** a ALBA Synchrotron Light Facility, Carretera BP 1413, Km. 3.3, Cerdanyola del Vallès, Barcelona 08290, Spain

**Keywords:** spectromicroscopy, X-ray absorption, photoemission, LEEM, PEEM, X-ray magnetic dichroism

## Abstract

The spectroscopic LEEM-PEEM experimental station at the CIRCE helical undulator beamline, which started user operation at the ALBA Synchrotron Light Facility in 2012, is presented. This station, based on an Elmitec LEEM III microscope with electron imaging energy analyzer, permits surfaces to be imaged with chemical, structural and magnetic sensitivity down to a lateral spatial resolution better than 20 nm with X-ray excited photoelectrons and 10 nm in LEEM and UV-PEEM modes. Rotation around the surface normal and application of electric and (weak) magnetic fields are possible in the microscope chamber. *In situ* surface preparation capabilities include ion sputtering, high-temperature flashing, exposure to gases, and metal evaporation with quick evaporator exchange. Results from experiments in a variety of fields and imaging modes will be presented in order to illustrate the ALBA XPEEM capabilities.

## Abstract 2

The performance of the spectroscopic LEEM-PEEM experimental station at the ALBA Synchrotron is presented: XPEEM lateral resolution down to better than 20 nm, electron energy resolution below 0.15 eV, and excellent long-term stability.

## 1. Introduction   

The ALBA XPEEM experimental station is based on a Spectroscopic PhotoEmission and Low Energy Electron Microscope (SPELEEM from Elmitec GmbH) permanently installed at the CIRCE beamline at the ALBA Synchrotron Light Facility, close to Barcelona (Spain) (http://www.cells.es/en). The beamline, based on a collimated-light plane-grating monochromator (Follath & Senf, 1997 ▶), provides soft X-ray photons in the 100–2000 eV energy range with high photon energy resolution and intensity, and full polarization control thanks to the APPLE II helical undulator used as source (Sasaki, 1994 ▶). It has been open to external users and in continuous improvement since October 2012. Beam time at CIRCE is allocated to academic users worldwide *via* peer-reviewed proposals submitted through the ALBA web portal (http://useroffice.cells.es/). Beam time is also available to industrial users.

In the following, we present the characteristics and features of the experimental station, briefly describe the different operation modes of the microscope, and show results representative of the performance demonstrated so far.

## 2. The experimental station layout   

**Figure 1:**
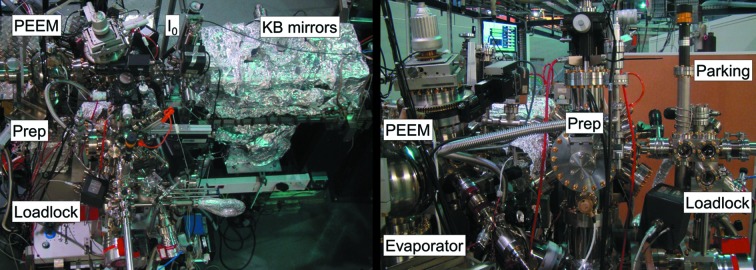
Left: top view of the experimental station, I_0_ and KB refocusing mirrors chambers. Right: side view of the experimental station comprising the PEEM main chamber with evaporator ports, preparation chamber, entrance chamber with sample parking and fast pumping load-lock.

The experimental station is formed by five separable ultra-high-vacuum sections: load lock, entrance chamber, preparation chamber, main chamber and electron optics. The load lock can be evacuated from atmosphere to 10^−6^ mbar in less than half an hour, so that a sample can be introduced from air to the main chamber in less than one hour. The entrance chamber is an intermediate section between the load lock and the preparation chamber, containing a parking for up to five samples and a docking station for a vacuum suitcase used to transfer samples from and to other stations and preparation chambers. General views of the experimental station are shown in Fig. 1 ▶.

An I_0_ measurement chamber is installed directly upstream of the microscope, equipped with fluorescent screen, gold mesh, gold foil, gold evaporator and calibrated photodiode. However, in most cases the normalization signal is extracted directly from the drain current of the last focusing mirror.

Since the emergent synchrotron photon beam is always wider in the horizontal than in the vertical, and the microscope geometry imposes a 16° near-grazing incidence, the microscope chamber is oriented such that the sample surface is nearly horizontal (face-down), and thus the elongation due to the beam projection on the surface is along the narrow vertical dimension of the photon beam. A detailed finite element analysis was performed in order to optimize the microscope support in this unconventional geometry for minimum vibrations. An added advantage is an enhanced sample manipulator stability with respect to other setups with vertical sample surface.

**Figure 2:**
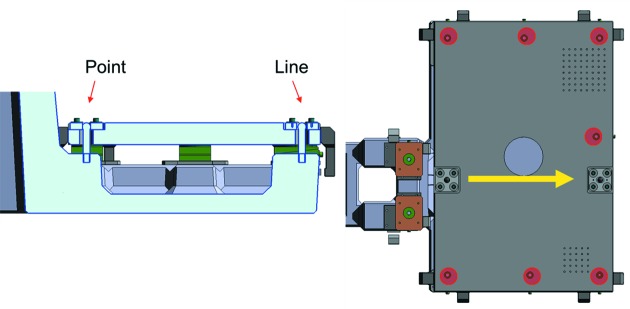
Link between Al baseplate and granite support. The fixations marked with red circles are loosened to allow expansion during bake-out, while the point and line interfaces ensure position recovery.

The microscope, I_0_ and refocusing mirrors chambers are all mounted on a common synthetic granite support, designed to maximize rigidity while minimizing weight and thus low-frequency vibration modes. In this way, any position drift of the experimental station with respect to the source will be de-magnified by the same factor as the photon beam. The maximum demagnification reachable with the bendable re­focusing Kirkpatrick–Baez (KB) mirror pair (Kirkpatrick & Baez, 1948 ▶) is 3.8:1 and 6.1:1 in the horizontal and vertical planes, respectively. The microscope chamber assembly itself is installed on the standard Elmitec aluminium baseplate and can be aligned with respect to the granite by a combination of precision levelers (AirLoc Schrepfer) and pusher elements. A specially designed link, visible in Fig. 2 ▶, avoids deformations due to differential expansion during full system bake-outs by permitting expansion of the Al plate and ensuring that it returns to the same position within less than 100 µm. The fine beam steering is performed with the refocusing mirror mechanics.

**Figure 3:**
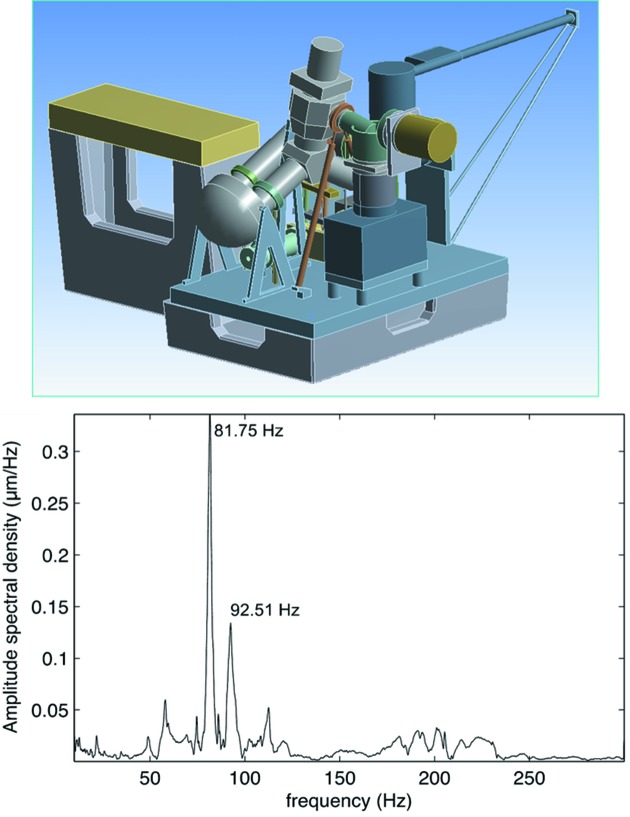
Top: model used for the vibration analysis of the experimental station and its support structure, after optimization. Bottom: eigen-frequency spectrum measured at the microscope sample manipulator.

A simplified but quite accurate model of the full assembly was used to simulate the vibrational eigenfrequencies (Fig. 3 ▶, top). After installation, the resonance frequencies of the PEEM sample manipulator (top-most component) were obtained by measuring the spectrum of its response to a hammer shock with a Renishaw ML10 interferometer and a reference suspended on an elastic support. The interferometer reference vibrates at a frequency below 5 Hz, and attenuates by a factor of 10^3^ any oscillation above 10 Hz. As can be seen on the graph in Fig. 3 ▶, the main resonance modes appear at 81.75 Hz and 92.51 Hz. Thanks to the absence of low-frequency resonances, the background mechanical noise at the manipulator is very low. The measured amplitude of the total oscillation, integrating for all frequencies above 10 Hz, is 18 nm r.m.s., similar to what was measured directly on the floor of the experimental hall.

## 3. The microscope   

In addition to the beamline soft X-rays, enabling X-ray photoemission electron microscopy (XPEEM), the microscope has a LaB_6_ electron gun for low-energy electron microscopy (LEEM) and diffraction (LEED) and a mercury lamp for UV-PEEM.

In LEEM, electrons back-scattered at the sample surface are used to form a magnified image, providing surface structural characterization in real time with 10 nm lateral resolution (Bauer, 1994 ▶). In PEEM, electrons emitted after excitation with a photon source are collected to form the image (Harp & Tonner, 1988 ▶). The image formed by the electron-optical lens system is intensified by a micro-channel plate and finally detected on a phosphor screen by an in-air CCD camera. For a comprehensive review of surface microscopy with low-energy electrons, see Bauer (2014 ▶).

XPEEM in combination with the high-brightness, tunable photon energy, and polarization of the CIRCE beamline provides a variety of laterally resolved operation modes based on X-ray absorption and photoelectron spectroscopy, the latter thanks to the imaging electron energy filter included in the imaging column. Along with chemical contrast mechanisms, the fully variable polarization makes magnetic imaging possible *via* the X-ray magnetic circular and linear dichroism effects (XMCD and XMLD) at the appropriate absorption edges of ferromagnetic and antiferromagnetic materials (Anders *et al.*, 1999 ▶). The total electron kinetic energy resolution in photoemission mode is determined by the combination of the resolution of the hemispherical electron energy analyzer (down to below 0.15 eV) and that of the beamline, usually negligible in comparison with that of the analyzer. In X-ray absorption mode, the energy resolution is solely determined by the beamline resolving power *E*/Δ*E*, which is tunable up to 8000 throughout the photon energy range.

**Figure 4:**
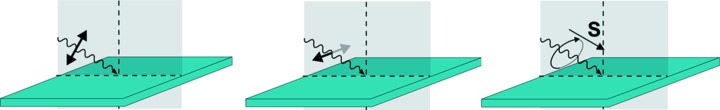
Relative orientation of polarization and sample for vertical, horizontal and circular polarization.

The orientation of the photon beam polarization with respect to the sample for vertical, horizontal and circular polarizations is sketched in Fig. 4 ▶. As can be seen, linear polarization can be either fully in the surface plane (horizontal polarization) or mostly out of plane (vertical polarization). Since the manipulator can be rotated around the sample normal (azimuthal rotation), and linear polarization with intermediate angles can be provided by the undulator, any relative orientation of the photon electromagnetic field and the sample can be reached in linear polarization up to 74° out of plane. In circular polarization, used for magnetic dichroism, the maximum contrast is obtained for in-plane magnetization, with a small out-of-plane component (16° grazing incidence). XMCD at different azimuthal angles enables vectorial magnetometry in order to fully characterize the magnetization vector.

An additional operation mode consists of microspot X-ray photoemission spectroscopy (µ-XPS), where a micrometre-sized area is selected by an aperture placed at an image plane of the microscope. The energy-dispersive plane of the analyzer is projected onto the detector, so that a photoemission spectrum originating from the selected region is recorded in a single shot, thus enabling real-time micro-spectroscopy. The spectrum width is around 10 eV with a resolution below 0.15 eV.

Using X-ray illumination and the lenses in diffraction mode, photoelectron diffraction can be performed, imaging several Brillouin zones at once for a chosen electron kinetic energy. This method is particularly useful for exploring the valence band dispersion of micrometre-sized areas. Details of the microscope electron optical layout and working modes can be found in Schmidt *et al.* (1998 ▶).

In standard operation conditions, the sample is at 20 kV and 2 mm away from the objective lens. A reduced field mode (15 kV at the same working distance) is available for special samples and/or sample holders with increased discharge risk, such as insulating samples, surface electrodes, degassing surfaces, *etc*.

The microscope lenses and CCD detector are integrated at the software level in the ALBA beamlines control system, based on Sardana, a supervision, control and data acquisition (SCADA) package inspired by the TANGO collaboration (Coutinho *et al.*, 2011 ▶). The integration of the microscope in the beamline control system allows for easy-to-use and flexible data acquisition macros.

## 4. The sample environment   

The preparation chamber is equipped with a four-axes manipulator, including heating and temperature measurement capabilities, sputter gun, precision gas dosing valves, and a port for fast evaporator exchange. It also contains many spare ports for the installation of extra equipment. The base pressure is below 10^−10^ mbar, but up to 10^−3^ mbar of inert gases can be dosed. In the main or microscope chamber the typical pressure is in the low 10^−10^ mbar and gases can be dosed at pressures below 10^−5^ mbar, limited by arcing due to the high voltage between sample and objective lens during imaging.

The microscope chamber also has heating and temperature measurement capabilities, two fast exchange evaporator ports and a few spare ports pointing at the sample position.

The standard Elmitec sample holder has four contacts electrically insulated from the sample potential, typically used for a filament and a W/Re thermocouple. With this holder, electron bombardment is used to heat the sample with up to 150 W power, reaching temperatures up to 2000 K and above for short periods of time. The maximum temperature during imaging is limited by thermal drift and sample degassing to about 1600 K.

Sample cooling with liquid nitrogen is possible in the microscope, although indirectly through sapphire crystals providing the necessary high-voltage electrical insulation. 150 K is easily reached after a couple of hours and lower temperatures (>100 K) should be reachable with appropriate LN_2_ pressure and flow. However, as usual in this kind of instrument, the need to control thermal drifts and vibrations from liquid-nitrogen evaporation implies considerable stabilization time (up to a few hours) and somewhat degraded spatial resolution.

Sample holders with two additional electrical contacts (six in total) for special setups are available and usable both in the preparation and main chamber manipulators. These contacts are used for example in combination with a custom-built electronic module (integrated in the high voltage rack) which permits applying electrostatic voltages up to ±240 V at the sample, as well as measuring the sample resistance. A basic current source is also available and there is sufficient free space inside the high-voltage rack for additional user developments. The sample holder contacts can be tested after sample mounting on an external test bench prior to introduction into vacuum. The same test bench is used to pre-align the sample tilt using the reflection of a diode laser.

**Figure 5:**
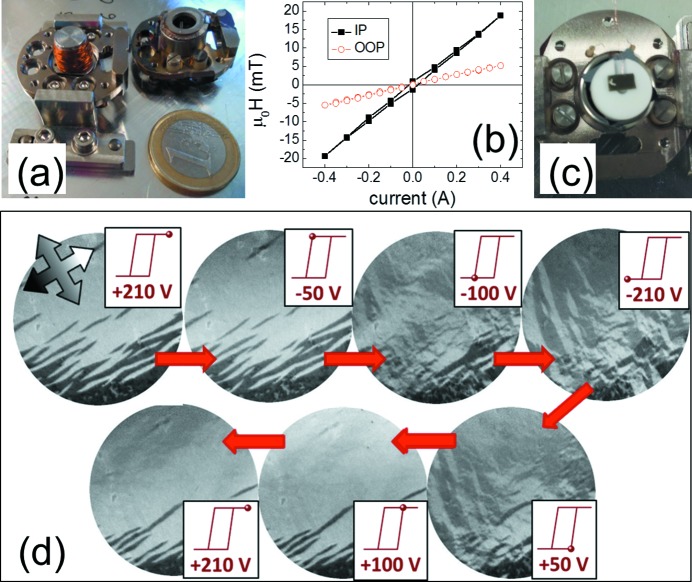
(*a*) Original Elmitec sample holder without cap (right) and sample holder with integrated out-of-plane electromagnet placed on the test bench. (*b*) Magnetic field produced at the sample position with the in-plane electromagnet sample holder. (*c*) Experimental realisation of out-of-plane electrical poling. The sample is mounted on an insulating spacer with a cut-out for wire connection. On the left of the sample surface some silver paint is visible that was used to assure good electrical contact with the top cap (not shown). (*d*) XMCD images of an LSMO thin film on a piezoelectric substrate with different applied voltages, showing hysteretic behaviour (Pesquera *et al.*, 2013 ▶). The piezoelectric substrate responds to the applied voltage by changing its lattice size and the strain transmitted to the thin film results in a modified magnetic domain structure due to the magnetoelastic effect or inverse magnetostriction. Field of view is 50 µm.

Special sample holder versions have been built for the *in situ* application of reversible in-plane and out-of-plane magnetic fields [the former adapted from the design of Kronast *et al.* (2010 ▶)], as well as electrostatic poling (Fig. 5 ▶). Sample holder electromagnets are powered by the standard filament current supply through a switching unit allowing spike-free current direction reversal, achieved by connecting a bypass between the power supply outlets (with a small resistance) while the polarity is switched. The magnetic fields applied at the measurement position are currently limited to about 20 mT, while in the sample parking it is possible to apply up to 200 mT in any in-plane direction using in-air permanent magnets. An adapter sample holder for in-vacuum transfer of samples to other beamline experimental stations and other setups at collaborating institutes is available to accommodate Specs- or Omicron-type sample plates.

Further developments in progress include biaxial in-plane magnetic fields, high-frequency cabling for the application of short pulses, and sample drain current measurements for integral absorption spectra.

## 5. Performance   

### 5.1. Beam size and stability   

For a 20 µm exit slit of the monochromator, the measured minimum spot size (attained with the smallest bending radius of the refocusing mirrors) is 3.2 µm × 35.6 µm FWHM (V × H), corresponding to a footprint on the sample of 11.6 µm × 35.6 µm FWHM (V × H). For most measurements the photon energy resolution attained with 100 µm exit slit (*E*/Δ*E* ≃ 6000) is more than enough, in which case the footprint on the sample is around 50 µm × 36 µm FWHM (V × H). Thus, fields of view of up to 50 µm-diameter can be imaged with good enough illumination homogeneity to obtain a flat background in differential imaging (such as XMCD-PEEM) without need for defocusing the KB mirrors. The beam position stability at the sample is sub-micrometre both in the vertical and horizontal directions over several hours.

**Figure 6:**
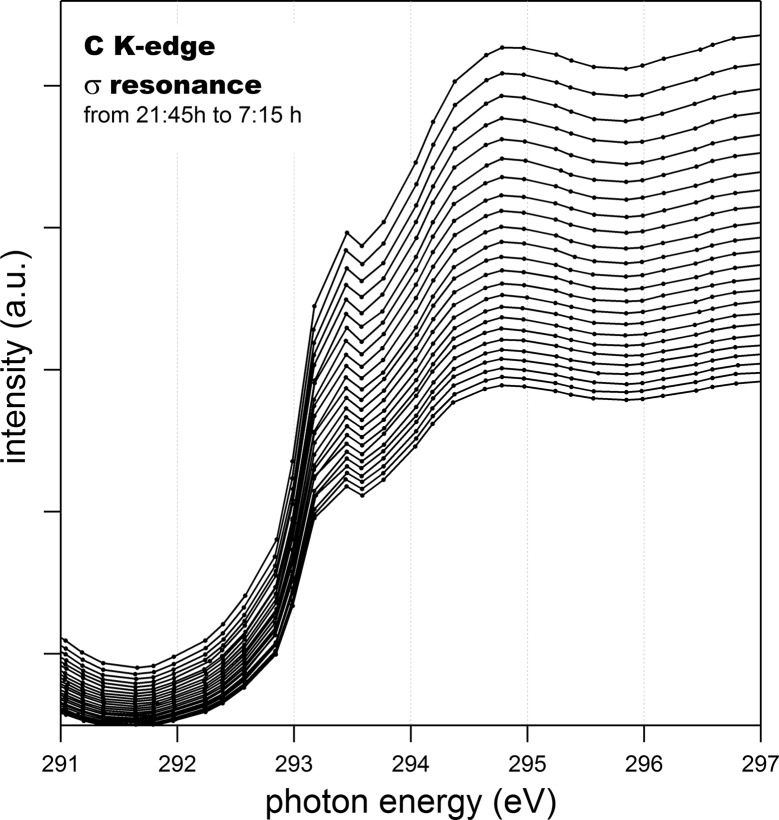
Detail of consecutive carbon *K*-edge absorption scans at the sigma resonance region over more than 9 h. The intensity reflects the ring current decrease since it was measured when ALBA operated in decay mode. The sample is highly oriented pyrolytic graphite.

The high beam stability in terms of position and energy permits measuring for many hours without the need for readjustment. In fact, no energy shift can be detected over many hours with the typical energy step of 0.1 eV used in PEEM experiments. In Fig. 6 ▶, consecutive absorption C *K*-edge scans over almost 10 h are plotted to illustrate the photon energy stability. This corresponds to the worst case in terms of thermal load on the optics due to the low photon energy and the fact that scans were started immediately after opening the beamline front end. At present, ALBA is operating in top-up mode and we are thus confident that thermal effects will be negligible even as the operation current of the ring is increased.

### 5.2. Microscopy   

**Figure 7:**
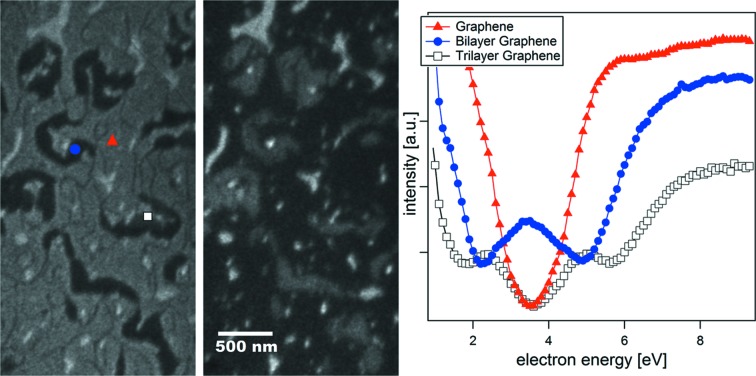
LEEM images at different electron energies and *I*(*V*) curves of graphene prepared *in situ* in the microscope chamber by annealing SiC up to 1550 K (Merino *et al.*, 2013 ▶). As can be seen, graphene regions of different thickness (namely single-, double- and triple-layer) have each a characteristic low-energy electron reflectivity curve. Regions with different graphene thickness can thus be distinguished in the LEEM images by their relative intensity.

The highest spatial resolution is obtained in LEEM mode, where a lateral resolution below 10 nm can be achieved, which corresponds to the state-of-the-art for a non-aberration-corrected instrument. In the vertical direction, contrast can reach the atomic level thanks to phase contrast at steps or quantum size effects in ultrathin films (Altman *et al.*, 1998 ▶). For example, graphene with different number of layers can be easily distinguished due to its characteristic reflectivity as a function of electron energy, as shown in Fig. 7 ▶.

**Figure 8:**
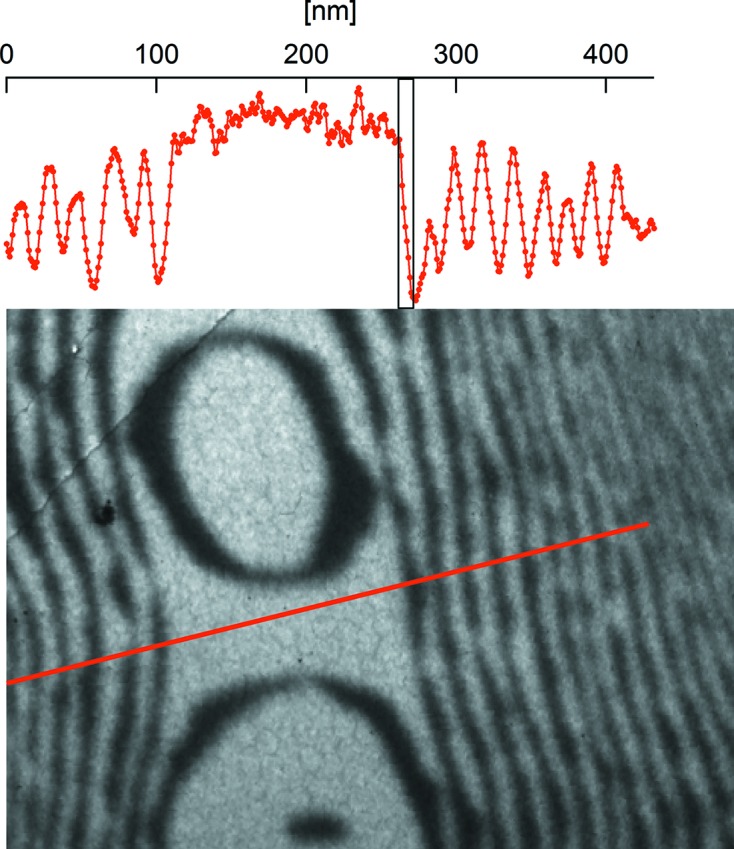
Dark-field LEEM image at 4.6 eV electron energy on one of the reconstruction beams of the clean Si(100)-2 × 1 surface (top) and line profile across several atomic steps. A 10 nm-wide bar is included in the profile graph as a guide to the eye. A line in the image indicates the profile direction.

Fig. 8 ▶ depicts a micrograph of the Si(100)-2 × 1 surface acquired in dark-field LEEM mode. In this mode a diffracted electron beam is used to form the image. In this case, using the (0, 1/2) beam, atomic terraces with the two orthogonal domains of the 2 × 1 reconstruction are alternatively bright and dark. As can be seen in the top profile, the lateral resolution is about 8 nm (15–85% edge jump).

**Figure 9:**
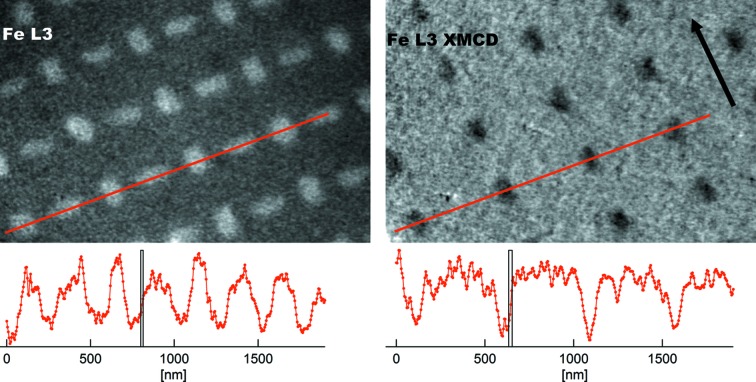
Direct XPEEM and XMCD-PEEM images of permalloy nanostructures on a Si substrate: elemental and magnetic contrast. Profiles along the highlighted lines are shown below each graph including bars of 15 nm (left) and 20 nm (right) width, as guides to the eye. The measurements were made in remanence after magnetically saturating the sample. Due to shape anisotropy, all nanostructures are magnetized along their long axis. In the XMCD image, nanostructures with magnetization perpendicular to the X-ray incidence (indicated by a black arrow) are not visible while those with anti-parallel magnetization appear dark. For a review on magnetic nanostructures see Martín *et al.* (2003 ▶).

When imaging with photoelectrons, resolution is reduced with respect to LEEM due to two main factors: chromatic aberrations (mainly of the objective lens) and lower signal, leading to longer exposure times and worse statistics. Further experimental factors can affect spatial resolution such as photoemission cross-section, sample conductivity or sample drifts due to thermal variations. In Fig. 9 ▶, the XPEEM lateral resolution for both elemental and magnetic imaging is illustrated for permalloy nanostructures on a silicon substrate, measured at room temperature. The nanostructures are 80 nm × 200 nm × 30 nm (w × l × h) and have been previously magnetized in one (in-plane) direction. However, in remanence they are magnetized along their respective long axis due to shape anisotropy. Thus, while all nanostructures are visible in the chemical map (at the Fe *L*_3_ absorption edge), only those with in-plane magnetization axis along the X-ray incidence axis are visible in the difference image of opposite circular polarizations (XMCD-PEEM). In the present case, their magnetization is antiparallel to the spin direction of the incident photon beam and they thus appear dark. The average edge width (15–85% step intensity) is 15 and 20 nm, respectively, to our knowledge the best demonstrated so far in non-aberration-corrected PEEM instruments.

### 5.3. Spectroscopy   

**Figure 10:**
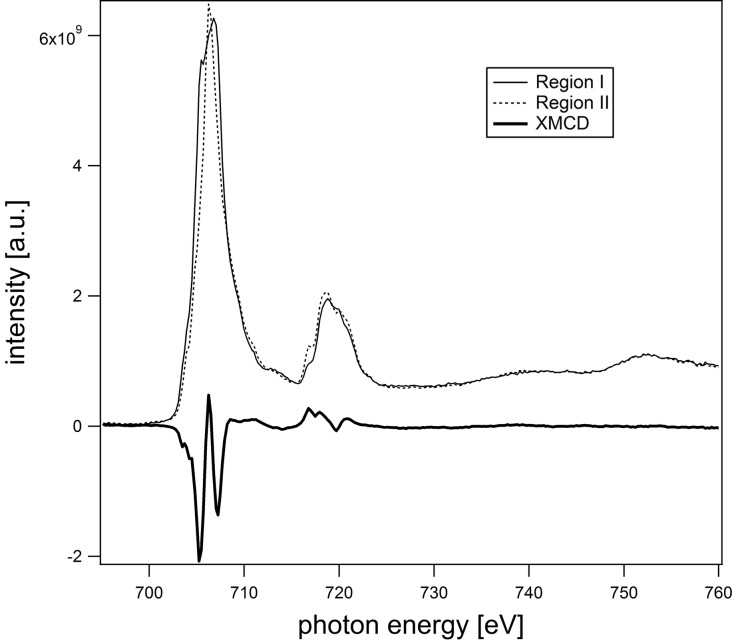
XMCD spectra of two different Fe_3_O_4_ domains and the resulting dichroic difference spectrum (Martín-García *et al.*, 2015*a* ▶).

The full strength of the XPEEM is based on the combination of imaging and full spectroscopic capabilities. In spectro-microscopy, a stack of images is acquired while varying either the photon energy (absorption or XAS mode) or the photoelectron kinetic energy (photoemission or XPS mode). In this way, spectra can be obtained from individual regions within the field of view, down to the pixel level. For example, combining XAS and circular polarization, an XMCD spectrum can be obtained by simply comparing the spectra of two oppositely magnetized regions or by comparing the spectra of a single region with two opposite helicities of the photon beam. In practice, the average of both is used in order to minimize experimental artifacts. In Fig. 10 ▶, spectra for two regions with opposite magnetization on the surface of a single-crystalline magnetite sample (Fe_3_O_4_) are shown together with their difference. The beam incidence was along a magnetic easy axis, the acquisition time was 1 s per image, and the regions used for extracting the spectra were about 2 µm × 2 µm. As can be seen, both the direct spectra and the dichroic difference have excellent quality.

**Figure 11:**
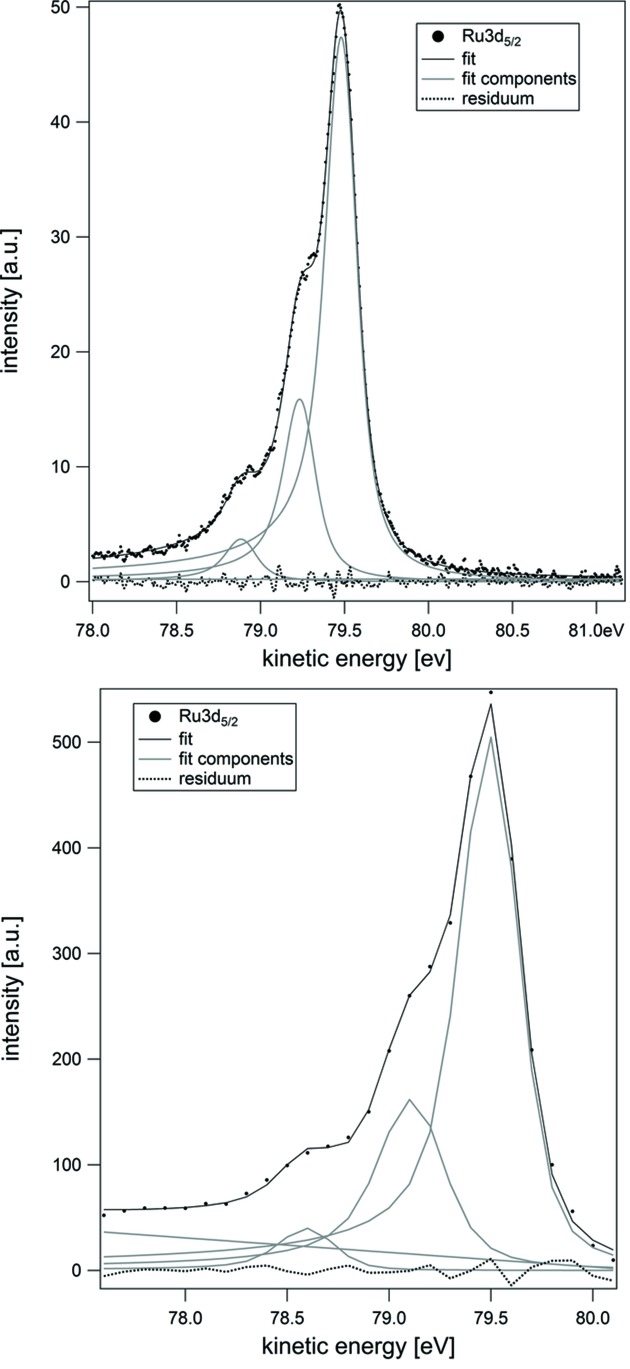
Ru 3*d*_5/2_ core-level µ-XPS spectrum from a 0.5 µm-diameter selected area (top) and the same spectrum extracted from a spectro-microscopy stack (bottom). The sample is an oxygen-covered Ru(0001) surface [p(2 × 2)-3O reconstruction (Martín-García *et al.*, 2015*b* ▶)].

While in absorption mode the photon energy resolution directly corresponds to that of the beamline, in photoemission mode the limiting contribution stems from the microscope’s photoelectron energy analyzer. The maximum resolution is achieved in micro-spot spectroscopy mode, where a small sample area is selected in an image plane by means of an aperture, and the dispersive plane of the analyzer is projected onto the detector. In this way, spectra from areas down to 0.5 µm in diameter can be measured, with an energy resolution down to almost 0.1 eV. Fig. 11 ▶ (top) is an example of such a µ-XPS spectrum and its fit, with Gaussian widths of 0.11 eV.

In imaging mode, a slit is inserted at the above-mentioned dispersive plane of the analyzer in order to limit the energy spread of the electrons used to form the image. As seen at the bottom of Fig. 11 ▶, in this mode the best energy resolution achieved so far with reasonable intensity is 0.25 eV while the expected limit value is slightly below 0.2 eV.

Both X-ray absorption measured *via* electron yield and photoelectron spectroscopy are surface-sensitive techniques. Selecting the appropriate photoelectron kinetic energy with the energy analyzer one can choose the desired photoelectron escape depth and thus vary the surface sensitivity between about 5 nm and 0.5 nm.

### 5.4. Diffraction   

**Figure 12:**
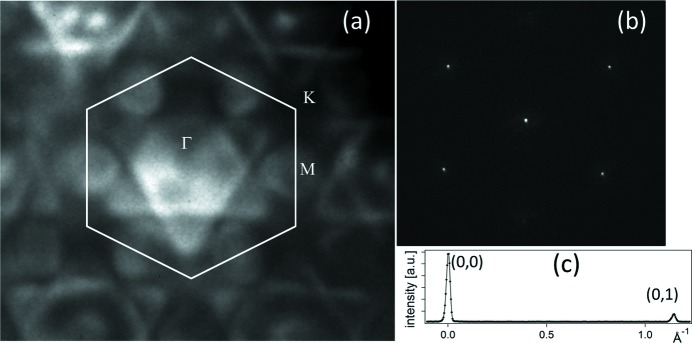
(*a*) Photoelectron diffraction pattern at the Fermi energy of a Ru(0001) surface. The first Brillouin zone and major symmetry points are indicated (Martín-García *et al.*, 2015*b* ▶). (*b*) LEED pattern of clean W(110) at 50 eV (5 µm electron beam diameter) and (*c*) profile across the (0,0) and (0,1) spots.

Selecting a small sample area by means of an aperture in an image plane, such as is done for micro-spot photoelectron spectroscopy (see §5.3), and setting the microscope electron-optical system in diffraction mode, one can obtain energy-filtered photoelectron diffraction patterns, with the same energy resolution as available in spectro-microscopy. In this way, either core-level photoelectron diffraction or valence band dispersions are measurable. Fig. 12(*a*) ▶ shows an example of a valence band diffraction pattern of a single Ru(0001) terrace (5 µm diameter area), at the Fermi energy. The full *k*-space half-sphere is recorded in a single shot, with a wavevector resolution of 0.1 Å^−1^ at 140 eV.

In the same lens mode but illuminating with the electron gun, micro-spot LEED is performed. Under the best conditions, *i.e.* restricting the electron illumination to a 0.5 µm-wide beam, we have determined a transfer width (or lateral spatial coherence) of 30 nm, while it is reduced to about 24 nm using an electron beam diameter of 5 µm. The transfer width is obtained by analyzing the spot profiles on a well known surface [for example, W(110) in Figs. 12(*b*) and 12(*c*) ▶].

## 6. Conclusions   

The spectroscopic LEEM-PEEM experimental station at the CIRCE beamline of the ALBA Synchrotron Light Facility is a versatile multi-technique instrument for state-of-the-art characterization of surfaces and thin films with a flexible sample environment. A variety of illumination and detection schemes permit a thorough structural, chemical and magnetic characterization in the same instrument with a switching time of minutes. The station has demonstrated excellent performance and is continuously evolving to accommodate user needs.
